# Effectiveness of Combination of Chitosan Gel and Hydroxyapatite from Crabs Shells (*Portunus pelagicus*) Waste as Bonegraft on Periodontal Network Regeneration through IL-1 and BMP-2 Analysis

**DOI:** 10.1155/2022/1817236

**Published:** 2022-03-20

**Authors:** Asdar Gani, Risfah Yulianty, Supiaty Supiaty, Machirah Rusdy, Gustivanny Dwipa Asri, Dian Eka Satya, Ayu Rahayu Feblina, Harun Achmad

**Affiliations:** ^1^Department of Periodontology, Faculty of Dentistry, Hasanuddin University, Makassar, Indonesia; ^2^Department of Pharmaceutical Science and Technology, Faculty of Pharmacy, Hasanuudin University, Makassar, Indonesia; ^3^Periodontal Specialist Dental Education Program, Faculty of Dentistry, Hasanuddin University, Makassar, Indonesia; ^4^Department of Pediatric Dentistry, Faculty of Dentistry, Hasanuddin University, Makassar, Indonesia

## Abstract

**Background:**

Periodontitis can be treated by regenerating periodontal tissue using a bone graft. Several natural materials such as chitosan and minerals such as hydroxyapatite can be developed to increase periodontal tissue regeneration. Chitosan has a high potential in healing wounds. Hydroxyapatite has excellent properties such as biocompatibility, osteoconductive, osteoinductive, and osteogenesis, making it an ideal material for soft and hard tissue regeneration. Chitosan and hydroxyapatite can be obtained from the shells of crustaceans, such as crabs shells (*Portunus pelagicus*).

**Objective:**

To assess the effectiveness of the combination of chitosan gel and hydroxyapatite powder as a bone graft on periodontal tissue regeneration in experimental animals. Periodontal tissue regeneration was assessed by expressing inflammatory cytokine gene indicators IL-1 and BMP-2.

**Methods:**

Experimental laboratory research and clinical trials with posttest only control group design. Twenty-seven Wistar rats were divided into three groups. Then the femoral bone defect was made, the positive control group was given placebo gel, the positive control group was given BATAN hydroxyapatite, and the test group was given a combination of chitosan gel and hydroxyapatite crab shells. Wistar rats were sacrificed on days 7, 14, and 21, and the femur bone was then taken for immunohistochemical analysis to determine the levels of IL-1 and BMP-2. The Kolmogorov–Smirnov test, Levene test, and one-way ANOVA analyzed the data.

**Results:**

On days 7, 14, and 21, the expression levels of IL-1 and BMP2 were significantly different between the three groups. The group added with chitosan gel and crab shell HA showed a faster decrease in IL-1 expression than the control group. BMP-2 expression increased in the test group compared to the control group.

**Conclusion:**

The combination of chitosan gel and hydroxyapatite inhibited the production of proinflammatory cytokines and increased the production of BMP-2.

## 1. Introduction

Periodontitis is an inflammation of the periodontal tissue caused by microorganisms, characterized by progressive loss of epithelial attachment, destruction of the periodontal ligament, destruction of alveolar bone, and pocket formation. The presence of an untreated pocket can lead to gingival recession and progressive alveolar bone resorption. Conditions like this can cause the tooth to pop out or fall out of the socket [1–3]. The National Institute of Dental and Craniofacial Research (NIDCR), the National Institutes of Health, United States, explains that there are almost 90% of the adult population over the age of 70 years experiencing periodontal-related diseases. Regeneration of the periodontal tissue is a continuous physiological process. Under normal conditions, new cells and tissues are constantly being formed to replace cells and tissues that have died [4]. When there is inflammation in the periodontal tissues, the first line of defense is activated. Macrophage infiltration as a protection against infection will also increase in the inflamed area and induce receptor activator kappa B (NFkB). NFkB will trigger the secretion of proinflammatory mediators, namely interleukin-1 (IL-1), interleukin 6 (IL-6), and tumour necrosis factor (TNF-*α*), to strengthen the immune response and accelerate metabolic processes. These proinflammatory mediators then regulate the receptor activator of NFkB ligand (RANKL) to bind to the receptor activator of NFkB (RANK), which causes increased differentiation of preosteoclasts into osteoclasts, then accelerates the process of bone resorption [5]. Osteoprotegerin produced by osteoblasts inhibits the development of osteoclasts. BMP-2 is a potent inducer of bone formation that promotes the differentiation of fibroblast cells into osteoblasts and chondroblasts. This BMP-2 increases callus formation in fracture healing [6].

Regeneration therapy accelerates healing and new bone formation. One type of tissue engineering to regenerate periodontally is applying bone grafts. There are four types of bone grafts, namely autograft, allograft, xenograft, and alloplastic synthetic material [7]. Autograft is still the primary choice in restoring bone defects but is still very limited, so replacement bone graft materials are needed to help bone regeneration. One of the readily available graft materials is xenograft, a natural material available in large quantities. Natural materials that have the potential as bone grafts in the regeneration and repair of bone tissue are chitosan and hydroxyapatite. Chitosan is a derivative of chitin, has osteoinductive properties, high biocompatibility, biodegradability, bioadhesion, antibacterial anti-inflammatory, and accelerates wound healing [8]. Hydroxyapatite is proven to be biocompatible and very well tolerated by human oral tissues, has osteoconductive ability, and has been shown to stimulate osteoblast differentiation and bone formation [9].

Research conducted by Shavandi et al. [10] showed that the combination of chitosan and hydroxyapatite has a scaffold pore size ranging from 90–220 m and has a porosity of 70–80%, indicating that the combination of chitosan and hydroxyapatite has physical and biological properties in the form of osteoconduction, osteoinduction, and osteogenesis. So that it can be a promising biomaterial for bone tissue regeneration when used as a bone graft [10]. The addition of chitosan into HA is expected to increase the effectiveness of HA in bioactive binding components and reduce HA fragility [11, 12].

One of the natural ingredients that contain chitosan and hydroxyapatite is crab shells. Waste from processing crabs shells, mostly shells; 70–80% are often discarded or only used as a mixture of animal feed, flavorings for making crackers, and shrimp paste. If this waste is left unchecked, it will cause environmental pollution and endanger human health. In general, crab shells contain protein (15.60%–23.90%), calcium carbonate (53.70%–78.40%), and chitin (18.70%–32.20%). Based on these contents, crab shell waste should not only be used to that extent because the waste is a source of energy potential that can be developed as bone graft material [13]. In this study, chitosan was used in the form of a gel. Gels have the benefit of remaining stable on the treated region and resisting evaporation for a longer period of time, allowing them to have their desired impact [14].

Therefore, this study aims to study the effectiveness of combining chitosan gel and hydroxyapatite from crab shell waste as a bone graft in periodontal tissue regeneration through IL-1 and BMP-2 analysis.

## 2. Materials and Methods

This type of research is actual experimental laboratory research. The research subjects were divided into three major groups, namely the group that was given a combination of chitosan gel and crab shell hydroxyapatite, the group that was given a placebo gel (negative control), and the group that was given BATAN's hydroxyapatite (HA) (positive control). Each group consisted of 9 Wistar rats. After adaptation to the laboratory environment for one week, the Wistar rats were then given a defect in the femur. First, anesthesia was performed using 0.1 ml ketamine injected at a dose of 0.11 ml/100 g body weight in the right femur (intramuscular). After the mice became unconscious, the fur to be made a defect was shaved. Next, antiseptic was applied to the area around the defect, and then a 2 cm incision was made using a blade on the soft tissue (skin and muscle) then removed with a periosteal elevator up to the femur. Then, the defect was made in a hole with a diameter of 5 mm and a depth of 1 mm using a round bur straight handpiece. Next, the negative control group was applied with placebo gel. Next, the second group was given the application of HA BATAN (positive control), and the third group was given the application of a combination of chitosan gel and crab shell hydroxyapatite (test group). After filling the defect, sutures are performed to close the femur extra. They were sacrificed then performed on days 7, 14, and 21 each of three rats. The samples were then analyzed using the IHC kit.

## 3. Results

**Figure 1:**
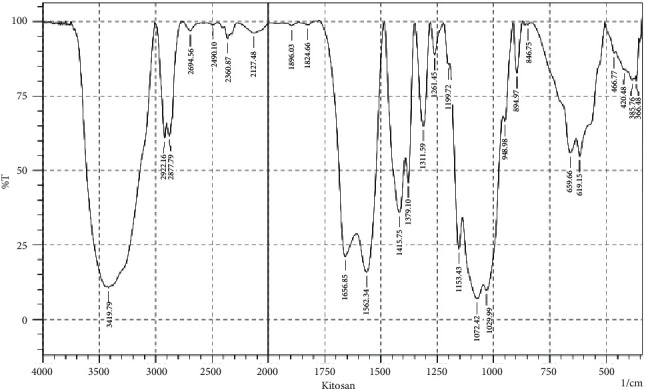
FTIR spectrum of crab (*portunus pelagicus*) shell chitosan gel.

After getting the results of the crab shell extract in the form of chitosan gel and hydroxyapatite powder, followed by the FTIR test (chitosan gel) and XRD test (hydroxyapatite powder). Examination of the content of this research material so that it is known for its natural content that is still awake.  Figure 1 shows the FTIR spectrum of chitosan from crab shells and squid bones in the 400–4000 cm^−1^ area; the absorption band at wave number 3419.79 cm^−1^ shows the OH and NH functional groups.

**Figure 2:**
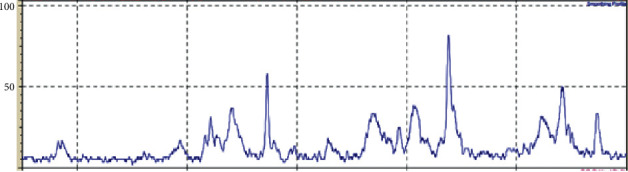
XRD spectrum of crab shell hydroxyapatite powder.

Figure 2 shows the XRD spectrum of the crab shell (*Portunus pelagicus*) hydroxyapatite powder. The peaks formed are HAp peaks. In the sample, the highest peak is 2*θ* = 67.2900. The grafting effectiveness of the combination of chitosan and hydroxyapatite gel from blue swimmer crab shells on IL-1 and BMP-2 was analyzed with a significance level of *p* ≤ =0.05 and the SPSS version 25.0 program.

The research data were analyzed descriptively to describe the distribution of the increase in data to clarify the results' presentation. First, the data obtained were tested for normality using the Kolmogorov–Smirnov Test, then the data was then tested for homogeneity using the Leaves Test. Finally, one-way ANOVA was used to analyze the differences between the research groups. The results of the analysis are declared significant, or there is a difference if *p* < 0.05.

On day 7, the mean BMP2 in the negative control group was 2.33 ± 0.577. The positive control group was 7.33 ± 1.527, and the mean BMP2 in the test group was higher at 11.33 ± 1.527. The statistical tests using one-way ANOVA showed a significant difference in the BMP2 value between the three groups with *p* ≤ =0.001 (*p* ≤ =0.05) (Table 1).

On day 14, the mean BMP2 in the negative control group was 4.67 ± 1.527; the positive control group was 7.67 ± 1.155. The mean BMP2 in the test group was higher at 13.00 ± 2.646. Statistical tests using one-way ANOVA showed a significant difference in BMP2 values between the three groups with *p* ≤ =0.005 (*p* < 0.05).

**Figure 3:**
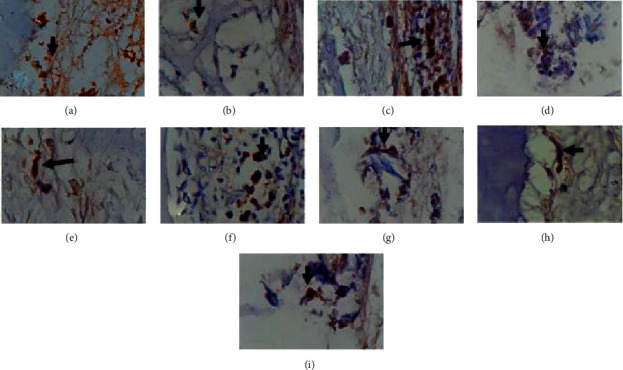
Expression of BMP-2 with 1000 times magnification, (a) expression of BMP-2 on the 7th day in the treatment group (a combination of chitosan gel and hydroxyapatite of crab shells), (b) expression of BMP-2 on the 14th day in the treatment group (combination of chitosan gel and hydroxyapatite of crab shells), (c) expression of BMP-2 day 21 in the treatment group (combination of chitosan gel and hydroxyapatite of crab shells), (d) expression of BMP-2 on day 7 in the control group positive (BATAN Hydroxyapatite), (e) 14th-day BMP-2 expression in the positive control group (BATAN Hydroxyapatite), (f) 21st day BMP-2 expression in the positive control group (BATAN Hydroxyapatite), (g) BMP-expression 2 days seven negative control group (placebo gel), (h) BMP-2 expression on 14 days negative control group (placebo gel), and (i) BMP-2 expression on 21 days negative control group (placebo gel).

On day 21, the mean BMP2 in the negative control group was 6.00 ± 2,000; in the positive control group was 8.67 ± 1.127. The mean BMP2 in the test group was higher at 15.00 ± 2,00. Statistical tests using one-way ANOVA showed a significant difference in BMP2 values between the three groups with *p* ≤ =0.035 (*p* < 0.05). The expression of BMP-2 can be seen from the examination using the immunohistochemical method using antibodies; it can be seen in Figure 3.

On the seventh day, the mean IL-1 value in the negative control group was 11.33 ± 0.577. The positive control group was 9.67 ± 1.527, and the mean IL-1 in the test group was lower at 6.00 ± 1,00. Statistical tests using one-way ANOVA showed a significant difference in IL-1 values between the three groups with *p* ≤ =0.008 (*p* < 0.05) (Table 2).

On day 14, the mean value of IL-1 in the negative control group was 11.00 ± 2,00; in the positive control group was 8.67 ± 1.52, and the mean IL-1 in the test group was lower at 5.00 ± 2,64. Statistical tests using one-way ANOVA showed a significant difference in IL-1 values between the three groups with *p* ≤ =0.035 (*p* < 0.05).

**Figure 4:**
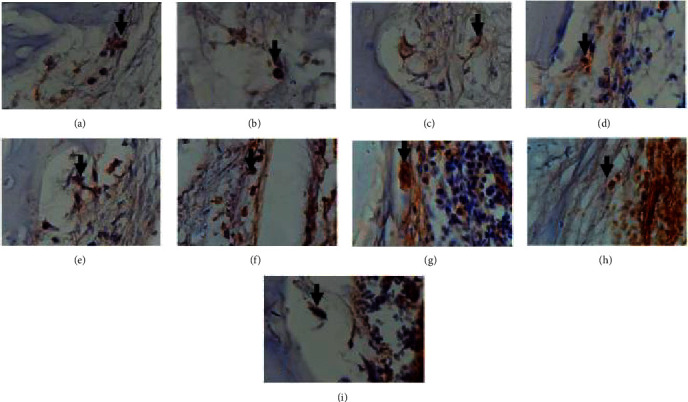
Expression of IL-1 with 1000 times magnification (a) expression IL-1 on the 7th day in the treatment group (a combination of chitosan gel and hydroxyapatite of crab shells), (b) expression of IL-1 on the 14th day in the treatment group (combination of chitosan gel and hydroxyapatite of crab shells), (c) expression of IL-1 day 21st in the treatment group (combination of chitosan gel and hydroxyapatite of crab shells), (d) expression of IL-1 on day 7th in the control group positive (BATAN Hydroxyapatite), (e) 14th-day IL-1 expression in the positive control group (BATAN Hydroxyapatite), (f) 21st-day IL-1 expression in the positive control group (BATAN Hydroxyapatite), (g) IL-1 expression 7th days in the negative control group (placebo gel), (h) IL-1 expression on 14th days negative control group (placebo gel), and (i) IL-1 expression on 21st days negative control group (placebo gel).

On day 21, the mean value of IL-1 in the negative control group was 8.67 ± 1.527; in the positive control group was 7.00 ± 2,00, and the mean IL-1 in the test group was lower at 3.67 ± 1,52. Statistical tests using one-way ANOVA showed a significant difference in IL-1 values between the three groups with *p* ≤ =0.035 (*p* < 0.05) (Figure 4).

## 4. Discussion

Chitosan's usage in surgery and periodontal/peri-implant care should not be overlooked. Chitosan has an antibacterial and antifungal action; therefore, it can help prevent infectious processes from developing in oral surgical wounds [15].

Based on the analysis results in Table 1 and Graph 1, it shows that between the control and test groups with observation days 7, 14, and 21 days of BMP-2 expression, an increase in BMP-2 expression was seen from day 7 to day 21. An increase in BMP expression-2 was seen in all groups, and there was a significant difference.

According to the researcher's analysis, the increase in BMP-2 expression is very relative depending on many factors, including surgical application technique and the nature of the material itself, in addition to the material's physical properties. The ability of the material to promote bone growth by allowing bone formation on its surface may be sufficient if sufficient quantities are required and bone margins are available. Based on the results of this study, the production of BMP-2 begins in the first week to week 2. However, it is increasingly seen that at the end of the third week, the production of BMP-2 increases; BMP-2 production may continue until the completion of the new bone tissue maturation phase.

Bone morphogenetic proteins are members of the transforming growth factor-beta family that play an essential role in osteogenesis. According to Al-Aql et al., the expression of BMP-2 increases at the beginning of the latent phase, possibly assisting the differentiation of precursor cells into chondrogenic or osteogenic cells [16]. The same thing said that Morphogen is an extracellular signal that regulates morphogenesis processes during mesenchymal-epithelial interactions [4].

Chenard et al. [17] studies of BMP expression in craniofacial skeleton fractures also confirms BMP involvement in the native craniofacial fracture healing process. In the absence of BMP-2 expression in nullizygous mouse limbs, bones are unable to initiate a regenerative response to fracture, while BMP-2 heterozygotes show a dose-dependent reduction in bone density.

Several studies have suggested that periodontal regeneration is mediated by Bone Morphogenetic Protein-2 and demonstrated that BMP induces osteogenic protein expression and promotes regeneration of bone and periodontal tissues, including cementum [18]. Abnormal levels of BMP-2 can cause congenital anomalies and associated disease. They are associated with mesenchymal cells that differentiate into muscle, fat, cartilage, and bone. The concentration of BMP-2 in specific tissues is essential because changes in levels can cause developmental anomalies or the development of various human diseases [19]. Min et al. [20] evaluated the use of chitosan-based hydrogels for bone morphogenetic protein-2 (BMP-2) delivery. The scientists claim that this chitosan gel can be employed for bone regeneration and repair because of its biocompatible and biodegradable qualities.

Autogenous bone is from the patient's iliac crest, tibia, mandible, or maxillary tuberosity, also known as rhBMP-2 (recombinant human bone morphogenetic protein-2). The capacity of rhBMP-2 to promote bone growth has been demonstrated as one of the most prevalent reconstruction procedures [21]. Many studies have evaluated the use of rhBMP-2 for preprosthetic maxillomandibular augmentation. Boyne et al. found that rhBMP-2 may be utilized to enhance the maxillary sinus floor for implant insertion in multicenter research. Jovanovic et al. found that the rhBMP-2Y-induced bone was not substantially different from the subject's native bone in a canine ridge augmentation paradigm. They found that rhBMP-2-induced bone was just as receptive to implant insertion and osseointegration as natural bone [22].

**Figure 5:**
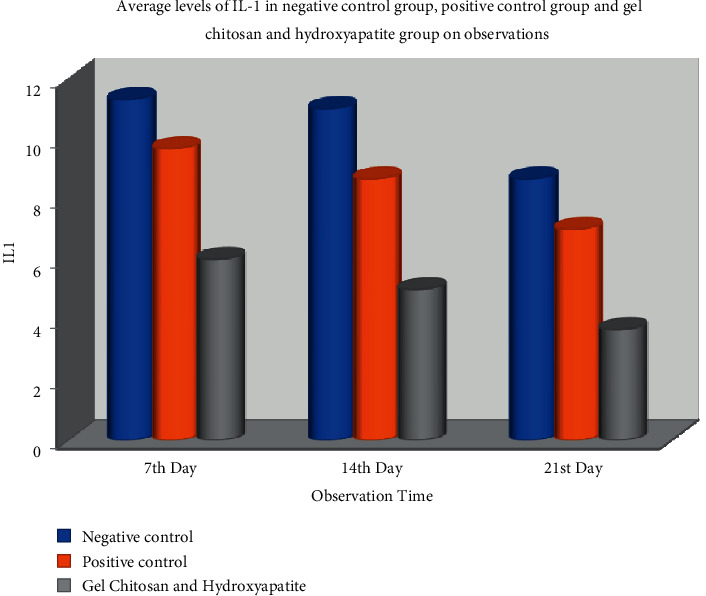
Bar chart of IL-1 expression between treatment group.

The results of the analysis in Table 2 and Figure 5 show that between the control group and IL-1 expression test in each group on days 7, 14, and 21 showed a significant decrease (*p* < 0.05) in IL-1 expression from day 7 to day 21. One thing to note is that the severity of inflammation varies between individuals, regardless of the degree of bacterial infection, suggesting that dysregulation of the inflammatory response host can contribute to the presence of microorganisms. Interleukin 1 is a cytokine that induces inflammation and plays a role in the atherosclerosis process so that it can be used as a target for antiatherosclerosis drugs [23].

Several studies have shown cytokines to be essential mediators associated with the pathogenesis of periodontitis. The complex network of cytokines that mediate the immune response includes proinflammatory cytokines, anti-inflammatory cytokines, and specific cytokine receptors. Cytokines are cell-specific biologically active molecules that elicit a specific response from other cells on which they act. Effective in low concentrations, produced locally in the tissue where it is produced. Inflammatory cytokines induced during the inflammatory response are closely related to the onset of their development. IL-1, IL-6, IL-8, and TNF- are classified as proinflammatory cytokines. Proinflammatory cytokines increase the bactericidal capacity of phagocytes, recruit additional innate cell populations to the site of infection, induce dendritic cell maturation, and subsequently direct specific immune responses against invading microbes [24].

The use of bone grafts from a combination of chitosan gel and hydroxyapatite of blue swimmer crab shells to replace bone structures that are lost or damaged due to defects after trauma or periodontal disease is expected to improve the wound healing process. In this case, chitosan and HA gels play an essential role in forming scaffolds, the attachment media for stem cells in bone defects [25]. Scaffolds also play an essential role in the initial extracellular matrix needed to assist cells in bone healing [26]. HA serves as a delivery medium for cytokines to bind and concentrate BMPs at the sites involved. HA also exhibits osteoinductivity properties. Osteoinduction occurs due to stimulation of the host stem cells. Activities: The activity of cell proliferation, differentiation, the metabolic activity of differentiated cells, and the release of calcium can stimulate osteoblast differentiation, thereby stimulating bone regeneration [27].

**Figure 6:**
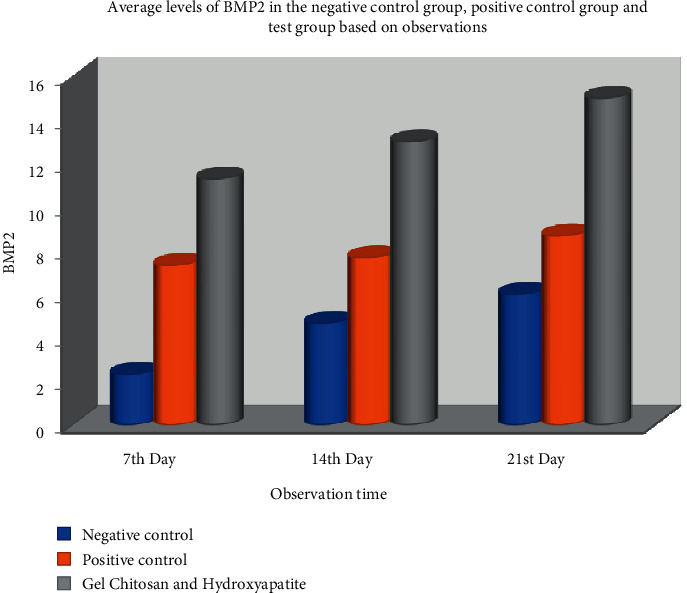
Bar chart of BMP-2 expression between treatment groups.

Figure 6 contains the Bar chart of BMP-2 expression between treatment groups.

The combination treatment group of chitosan and hydroxyapatite gel gave the most effective and significant results by showing the smallest average amount of interleukin compared to the control group. Likewise, the combination of chitosan gel and crab shell hydroxyapatite was most influential and significant by showing the highest average amount of bmp2 compared to the control group. Our research shows that these chitosan and hydroxyapatite products can reduce proinflammatory cytokines and shorten the inflammatory process [8].

The results of this study are in accordance with the results of research by Liao et al. [28], where mHA/chitosan scaffolds can inhibit the growth of periodontal pathogens. In vitro, composite scaffolds containing rhAm enhanced ALP activity and expression levels of RUNX-2, OPN, and DLX-5 genes and proteins. This research shows that tissue creation, such as periodontal regeneration, has antimicrobial properties and enhances osteogenic effects. In the future, we intend to use many waste crab shells to make chitosan with better characteristics, thereby helping to shorten inflammation and accelerate the proliferative and remodeling phases of bone formation.

## 5. Conclusion

Based on the results of this study, it can be concluded that there is a significant increase in the expression of BMP-2 and IL-1 in bone defects by administering a combination of chitosan gel and hydroxyapatite from crab shell *(Portunus pelagicus*) waste. The results of this study showed the inhibition of the production of the proinflammatory cytokine IL-1 and an increase in BMP-2, which is a marker of bone graft action as periodontal tissue regeneration. Further studies are needed with a larger study population to analyze the combination of chitosan gel and hydroxyapatite from crab shell *(Portunus pelagicus*) waste as a bone graft in periodontal tissue regeneration.

## Figures and Tables

**Table 1:** Descriptive statistics showing results of BMP-2 expressions.

Group	Sample	Day 7	Day 14	Day 21
		(Mean ± SD)	(Mean ± SD)	(Mean ± SD)
Chitosan gel and hydroxyapatite	9	11.33 ± 1.53	12.33 ± 2.08	15.00 ± 2.00
Positive control	9	7.33 ± 1.53	7.66 ± 1.15	8.66 ± 1.53
Negative control	9	2.33 ± 0.57	4.66 ± 1.52	6.00 ± 2.00
*p*		0, 001	0, 005	0, 003

**Table 2:** Descriptive statistics showing results of IL-1 expressions.

Group	Sample	Day 7	Day 14	Day 21
		(Mean ± SD)	(Mean ± SD)	(Mean ± SD)
Chitosan gel and hydroxyapatite	9	6.00 ± 1.00	6.00 ± 1.00	3.66 ± 1.53
Positive control	9	9.66 ± 1.53	8.66 ± 1.53	7.00 ± 2.00
Negative control	9	11.33 ± 1.53	11.00 ± 2.00	8.66 ± 1.53
*p*		0, 008	0, 035	0, 029

## Data Availability

The datasets generated during and/or analyzed during the current study are available from the corresponding author on reasonable request.
